# Performance of large language models in urological decision support: a guideline-based comparative evaluation in urolithiasis

**DOI:** 10.1007/s00240-026-02013-1

**Published:** 2026-06-06

**Authors:** Adem Tunçekin, Yasin Aktaş

**Affiliations:** Faculty of Medicine, Department of Urology, Uşak University, Uşak, Turkey

**Keywords:** Large language models, Urolithiasis, Clinical decision support, Guideline adherence, Artificial intelligence, Natural language processing

## Abstract

Large language models (LLMs) are increasingly investigated for their potential role in guideline-based clinical information support. However, their consistency with subspecialty guidelines, particularly in urolithiasis, remains underexplored. This study aimed to evaluate the performance of four large language models (LLMs); GPT-4, GPT-4-turbo, Claude, and Gemini in generating guideline-concordant responses to clinical questions related to urolithiasis. A total of 105 clinical questions were independently developed by the authors based on urolithiasis management principles. Each LLM generated responses in two separate sessions. Two experienced urologists evaluated the outputs for accuracy and concordance with guideline recommendations. Inter-rater agreement analysis demonstrated fair agreement between evaluators. Differences across models and guideline categories were assessed using appropriate statistical tests. All four LLMs demonstrated high guideline adherence, with mean total scores ranging from 86.5 ± 5.2 (Gemini) to 92.8 ± 3.1 (Claude). Claude achieved the highest correlation with expert ratings (*r* = 0.94, *p* < 0.01). There were no statistically significant differences across models or among the nine clinical categories (*p* > 0.05). Session-to-session repeatability was also high for all models, with intra-model correlation coefficients exceeding 0.90. LLMs, particularly Claude, can provide reliable, guideline-consistent answers to urolithiasis-related clinical queries. Their consistent performance across themes suggests utility as adjunctive informational tools for guideline-based urological education and support, although further validation in real-world clinical settings remains necessary.

## Introduction

Large language models (LLMs), including platforms such as GPT-4, GPT-4-turbo, Claude and Gemini, have rapidly emerged as tools with potential applications in healthcare decision-making. These models, trained on vast textual corpora, are capable of generating human-like responses to complex clinical prompts. As artificial intelligence (AI) continues to integrate into clinical workflows, evaluating the reliability and medical accuracy of these models has become increasingly critical [1, 2].

In particular, the role of LLMs in supporting adherence to established clinical practice guidelines has gained attention. Ensuring that AI-generated medical content aligns with evidence-based recommendations is essential to prevent misinformation and enhance clinical safety. Several recent studies have evaluated LLM performance across various medical domains, including oncology, internal medicine, and chronic disease management [3–5]. Moreover, early work within urology has assessed ChatGPT’s performance in testicular cancer and urolithiasis contexts [6–8]. However, comparative analyses across multiple LLM platforms and their agreement with expert assessments remain limited [9].

This study aimed to assess and compare the compliance of four widely used LLMs; GPT-4, GPT-4-turbo, Claude and Gemini with the European Association of Urology (EAU) guidelines for urolithiasis. By employing expert scoring and consensus methodology, we sought to determine the degree of alignment between AI-generated answers and evidence-based clinical standards.

## Methods

### Study design

This was a cross-sectional, comparative evaluation study designed to assess the compliance of large language models (LLMs) with the European Association of Urology (EAU) guidelines for urolithiasis. A total of 105 standardized clinical questions were developed by the authors, reflecting key recommendations based on EAU guidance. These questions were independently submitted to four state-of-the-art LLMs; GPT-4, GPT-4-turbo, Claude and Gemini.

The responses generated by each model were subsequently evaluated by two urologists with expertise in guideline-based clinical decision-making. Each response was scored on a 3-point ordinal scale: 0 = non-compliant, 1 = partially compliant, 2 = fully compliant, based on its alignment with the EAU guideline recommendations. In instances where the two urologists provided differing scores, a consensus was reached through joint discussion. This study did not involve any human subjects or identifiable patient data. Therefore, approval from an institutional ethics committee was not required.

## Large language models and question framework

The evaluation included four LLMs: GPT-4 and GPT-4-turbo (developed by OpenAI), Gemini (developed by Google DeepMind), and Claude (developed by Anthropic). All models were accessed between June 2025-September 2025 via their publicly available web interfaces under default settings. No fine-tuning or prompt engineering beyond the base query was applied. Each model generated responses during two separate sessions using identical question sets and standardized prompting procedures. The sessions were conducted independently under the same usage conditions in order to minimize contextual variability and evaluate response consistency across repeated interactions.

To ensure methodological consistency, all clinical questions were submitted using identical wording across all large language models. Each query was entered in a newly initiated chat session without follow-up prompts or conversational memory carryover. No additional contextual guidance, prompt engineering, or iterative clarification was provided. All models were evaluated using their publicly accessible web interfaces under default settings during the same study period.

The question set was developed by two urologists with clinical experience in endourology and guideline-based management of urolithiasis and categorized into 9 predefined thematic parts, each representing a distinct clinical domain relevant to urolithiasis. These parts included: [1] evaluation and diagnosis [2], acute management [3], ureteric stones [4], renal stones [5], pediatric stone disease [6], stone disease in pregnancy [7], infectious stones [8], metabolic evaluation, and [9] follow-up and recurrence. The categorization was not based on formal EAU guideline sections but was structured by the research team to reflect clinically meaningful groupings. Questions were derived from key diagnostic and therapeutic recommendations within the EAU urolithiasis guidelines, and efforts were made to ensure balanced representation of major guideline topics and varying levels of clinical complexity during question development. The full list of 105 clinical questions, categorized by urolithiasis-related themes, is provided in Supplementary Appendix A. These questions were used consistently across all evaluated LLMs. Although formal external validation or pilot testing was not performed, the study was designed as an exploratory comparative evaluation of guideline concordance rather than a formal psychometric validation study.

### Statistical analysis

All statistical analyses were conducted using IBM SPSS Statistics for Windows, Version 23.0 (IBM Corp., Armonk, NY, USA). Given the ordinal nature of the expert consensus scores, non-parametric tests were employed for all inferential procedures. The Friedman test was applied to assess overall performance differences among the four large language models across the full set of 105 questions. To investigate model performance variations across different thematic parts of the guideline-based question set, separate Kruskal–Wallis H tests were performed for each predefined category (*n* = 9). Spearman’s rank-order correlation coefficients were calculated to evaluate the degree of alignment between individual model scores and the final expert consensus. A two-tailed *p*-value < 0.05 was considered statistically significant in all analyses. Inter-rater agreement between the two independent expert evaluators was assessed using Cohen’s kappa coefficient prior to consensus resolution.

## Results

### Overall distribution of consensus scores across models

Descriptive statistics demonstrated that all four large language models achieved high levels of compliance with the EAU urolithiasis guidelines, with median consensus scores approaching 2.0. GPT-4-turbo recorded the highest mean consensus score (1.87 ± 0.34), while GPT-4 showed the lowest (1.81 ± 0.40), although overall variability among models was minimal. The Friedman test revealed no statistically significant difference in consensus scores among the models (χ² = 0.99, df = 3, *p* = 0.804), indicating a comparable level of performance in guideline adherence across GPT-4, GPT-4-turbo, Claude, and Gemini (Table 1).

**Table 1:** Descriptive statistics and overall comparison of question-level consensus scores between language models

Model	Mean ± SD	Median (IQR)	Min–Max	Friedman χ²	*p* value
GPT-4	1.81 ± 0.40	2.0 (1–2)	1–2	0.99	0.804
GPT-4-turbo	1.87 ± 0.34	2.0 (2–2)	1–2		
Gemini	1.82 ± 0.38	2.0 (1–2)	1–2		
Claude	1.86 ± 0.36	2.0 (2–2)	1–2		

SD: Standard deviation; IQR: Interquartile range; GPT: Generative Pretrained Transformer

There were no statistically significant differences in consensus scores across the four large language models based on the Friedman test (*p* = 0.804).

### Subgroup analysis by question categories

To explore whether the compliance of large language models (LLMs) varied across different domains of the EAU urolithiasis guidelines, a subgroup analysis was conducted based on predefined question categories (*n* = 9 parts). The Kruskal–Wallis H test was applied to compare consensus scores among the four LLMs; GPT-4, GPT-4-turbo, Claude, and Gemini within each question category. While minor variations in median scores were observed, none of the comparisons reached statistical significance (*p* > 0.05), indicating no significant differences in performance across the guideline domains (Table 2).

**Table 2:** Comparison of consensus scores among language models across question categories

Question Parts	χ² (df = 3)	*p* value
1. Evaluation and Diagnosis	1.406	0.704
2. Acute Management	0.934	0.817
3. Ureteric Stones	2.474	0.480
4. Renal Stones	1.240	0.743
5. Pediatric Stone Disease	1.230	0.745
6. Stone Disease in Pregnancy	1.066	0.785
7. Infectious Stones	0.805	0.849
8. Metabolic Evaluation	1.337	0.720
9. Follow-up and Recurrence	1.781	0.620

All Kruskal–Wallis tests were non-significant (*p* > 0.05), indicating no statistically meaningful variation in model performance across the question categories.

## Correlation between large language model consensus scores and expert agreement

Inter-rater agreement analysis demonstrated fair agreement between the two evaluators across all evaluated models prior to consensus resolution (Cohen’s κ range: 0.284–0.324, all *p* < 0.001). The analysis revealed strong and statistically significant positive correlations between the consensus scores generated by each large language model (LLM) and the expert-derived consensus ratings, reflecting a high degree of alignment. Among the models, Claude exhibited the strongest correlation with expert consensus (ρ = 0.964, *p* < 0.001), followed closely by GPT-4-turbo (ρ = 0.943, *p* < 0.001), GPT-4 (ρ = 0.938, *p* < 0.001), and Gemini (ρ = 0.862, *p* < 0.001). All correlations were statistically significant, indicating robust associations between each model’s outputs and expert judgment. Claude demonstrated the highest alignment, suggesting superior consistency with expert-level decision-making.

## Discussion

This study demonstrates that current large language models (LLMs) GPT-4, GPT-4-turbo, Claude, and Gemini are capable of generating responses with high adherence to the European Association of Urology (EAU) guidelines for the diagnosis and management of urolithiasis [10]. All four models achieved median consensus scores near full compliance, with no statistically significant differences among them. These findings highlight a key advancement in the ability of general-purpose LLMs to apply specialty-specific recommendations without explicit fine-tuning. Unlike traditional rule-based decision-support systems, LLMs rely on extensive pretraining across diverse data sources to generate clinically aligned content, offering potential utility for evidence-based informational support in clinical environments [11]. The present study should therefore be interpreted as an exploratory evaluation of guideline concordance rather than a direct assessment of autonomous clinical decision-making capability.

Our findings are consistent with previous studies that examined the performance of AI models in the context of urolithiasis, particularly ChatGPT. In a recent single-model analysis, Çakır et al. evaluated ChatGPT’s ability to answer both patient-oriented and guideline-based questions about urinary stone disease, reporting that 95% of the answers were completely accurate and clinically acceptable [7]. Their study highlighted ChatGPT’s strengths in general and diagnostic topics, as well as its capacity to reproduce consistent responses. However, their evaluation was limited to a single LLM version and did not involve direct model-to-model comparison or expert consensus scoring. In contrast, our study expands on this foundation by systematically comparing four leading LLMs and incorporating domain-expert validation to ensure methodological robustness.

Among the evaluated models, Claude demonstrated the highest correlation with expert consensus, suggesting a strong capability to generate guideline-consistent responses in the domain of urolithiasis. This performance may reflect architectural distinctions or training approaches that support structured medical information processing, as supported by recent evaluations showing Claude’s robust performance in diagnostic and decision-support tasks [12, 13]. The model’s relatively stable performance across various question themes may indicate potential utility in guideline-based informational support. Furthermore, the absence of significant differences in performance across the nine urolithiasis domains suggests that all LLMs evaluated in this study demonstrated relatively consistent performance across different thematic categories. This thematic stability may be relevant for future clinical and educational applications involving variable topic domains.

Despite the promising results, several limitations of this study should be acknowledged. The use of standardized questions derived directly from guideline content creates a controlled test environment that does not fully reflect the variability and complexity of real-world clinical interactions. In practice, clinicians must navigate incomplete data, patient-specific nuances, and diagnostic uncertainty—factors that may challenge the real-world applicability of LLM outputs. Moreover, although expert scoring strengthens the study’s validity, subjective interpretation during consensus formation may introduce bias. Recent evidence also shows that model behavior can vary significantly based on prompt phrasing and clinical context, raising concerns about consistency and stability in live decision-making environments [14, 15]. These considerations underscore the importance of cautious implementation and ongoing evaluation of LLMs in real-time clinical settings. Real-world clinical decision-making involves individualized patient characteristics, imaging interpretation, laboratory findings, and evolving clinical scenarios that cannot be fully represented through standardized guideline-based questions alone.

The results of this study underscore the emerging potential of LLMs as supportive tools in specialty-specific medical domains, particularly when benchmarked against established clinical guidelines. Their ability to generate accurate, consistent, and guideline-adherent responses suggests potential utility for future integration into clinical informational support systems. However, translating these capabilities into everyday practice will require further validation across diverse real-world scenarios, patient populations, and languages. Future research should focus on evaluating LLM performance within live clinical workflows, assessing user interaction dynamics, and exploring model integration with electronic health records. In parallel, regulatory frameworks and ethical standards must evolve to ensure responsible deployment, clinician oversight, and patient safety. With continued development, LLMs may become reliable assistants in urological care and beyond complementing but not replacing clinical expertise.

## Conclusion

This study shows that LLMs, particularly Claude, can provide clinically consistent answers to urolithiasis guideline-based questions. Claude demonstrated the highest alignment with expert consensus. While model performance was stable across themes, real-world application will require further validation. LLMs may serve as adjunctive informational tools for guideline-based urological education and support, although further validation in real-world clinical settings remains necessary.

## Data Availability

The datasets generated and analyzed during the current study are available from the corresponding author upon reasonable request.

## Appendix A

**Table:**

Appendix A. Full List of 105 Guideline-Based Clinical Questions Used for Large Language Model Evaluation
**QUESTIONS**
**1. Epidemiology & General Information (Q1–Q10)**
1. What is the general prevalence of kidney stones?
2. What is the prevalence of stones in males and females?
3. What is the prevalence of bladder stones in men?
4. In which age group are stones most commonly seen?
5. During which seasons does stone disease increase?
6. In which geographical regions is stone disease most common?
7. In which age group are bladder stones most common in children?
8. Has the prevalence of stone disease increased in recent years?
9. What is the most common cause of stone formation?
10. How important is genetic predisposition in stone disease?
**2. Metabolic / Biochemical / Genetic (Q11–Q25)**
11. What is the most common type of stone?
12. Which stones are radiolucent?
13. What causes struvite stones to form?
14. How are cystine stones identified?
15. What is primary hyperoxaluria?
16. Which drug is used in the treatment of hyperuricosuria?
17. What is hypercalciuria?
18. Which vitamin should be restricted in calcium stones?
19. At what urine pH do uric acid stones form?
20. Which stone type is suspected if urine pH is above 7.5?
21. What are examples of genetic stone diseases?
22. Which type of stone is associated with infection?
23. What is the first test for metabolic evaluation?
24. What is the inheritance pattern of cystinuria?
25. In which metabolic disorder is allopurinol used?
**3. Imaging & Diagnosis (Q26–Q36)**
26. What is the first-line imaging modality for kidney stones?
27. What is the preferred diagnostic method for stones in children?
28. What is the first choice for stone diagnosis during pregnancy?
29. What is the sensitivity of non-contrast CT?
30. Which radiolucent stones are not visible on CT?
31. When should imaging be performed for residual stones after ESWL?
32. What are the advantages of ultrasound in stone diagnosis?
33. Which imaging is required for anatomical evaluation before PCNL?
34. What is the preferred imaging method for lower pole stones?
35. Which imaging is used if uric acid stone is suspected?
36. Which emergency imaging is used in suspected stone complications?
**4. Pediatric Urolithiasis (Q37–Q46)**
37. What is the most common metabolic cause of stone formation in children?
38. What is the most common stone type in pediatric patients?
39. Is there a link between systemic disease and pediatric stone disease?
40. How do genetic disorders influence stone disease in children?
41. Who should undergo metabolic evaluation among pediatric patients?
42. When is ESWL contraindicated in children?
43. What is the most common symptom in children with stones?
44. What is the spontaneous stone passage rate in children?
45. What is the recurrence rate of stones in pediatric patients?
46. What criteria guide surgical intervention in pediatric stone disease?
**5. Medical Treatment (Q47–Q56)**
47. What is the first-choice analgesic in renal colic treatment?
48. Which drug class is used in MET therapy?
49. At what stone size is alpha blocker therapy effective?
50. What is the maximum recommended duration of MET therapy?
51. What are the failure criteria for MET?
52. What is the spontaneous passage rate of stones < 5 mm?
53. What is the recommended daily fluid intake to prevent stones?
54. What is the dietary recommendation in hypocalciuria?
55. What is the recurrence rate within 5 years after the first stone episode?
56. When is prophylactic antibiotic use indicated in stone management?
**6. Surgery & Interventions (Q57–Q71)**
57. What are the indications for ureteroscopy?
58. For which stone sizes is PCNL the first-line treatment?
59. What are the failure criteria for ESWL?
60. How effective is ESWL for lower pole stones?
61. What is the maximum stone size suitable for RIRS?
62. What are the advantages of mini-PCNL?
63. What is the most common complication after PCNL?
64. What factor reduces the need for DJS after URS?
65. Which technique reduces bleeding risk in PCNL?
66. What is the recommended follow-up duration after stone surgery?
67. What is the ideal patient position for PCNL?
68. What is steinstrasse?
69. What is the complication rate of URS?
70. How frequent is hematuria after ESWL?
71. Which is the least invasive surgical option for kidney stones?
**7. Special Patient Groups (Q72–Q81)**
72. What is the first diagnostic method during pregnancy?
73. Can ESWL be used in pregnancy?
74. Which trimester is safest for URS in pregnancy?
75. What is the most common stone type in SCI patients?
76. Why is the stone risk increased in neurogenic bladder?
77. What is the frequency of stones in transplant patients?
78. What is the stone formation rate after urinary diversion?
79. Where are stones most frequently located in transplant kidneys?
80. Which analgesics are preferred during pregnancy?
81. What should be considered when performing PCNL in diversion patients?
**8. Complications & Follow-up (Q82–Q92)**
82. What is the most common complication after URS?
83. What systemic complications may develop after ESWL?
84. What is the sepsis rate after PCNL?
85. How is bleeding managed after PCNL?
86. What is the post-operative follow-up algorithm?
87. How do subclinical infections affect stone treatment?
88. How long should antibiotic prophylaxis continue after surgery?
89. What is the first step in managing ureteral perforation?
90. What is the rate of stricture formation after URS?
91. When does a hematoma require intervention?
92. What is the most common device-related complication?
**9. Risk Factors & Others (Q93–Q105)**
93. Which diet increases stone risk?
94. What is the effect of a vegan diet on stone risk?
95. Do antacids increase stone risk?
96. How does a sedentary lifestyle influence stone risk?
97. How important is family history in stone disease?
98. Do occupations influence the risk of stone disease?
99. How do GI diseases influence stone formation?
100. What is the bladder stone rate in BPH patients?
101. What is the most common infection-related stone?
102. Does metabolic syndrome affect stone disease?
103. How much does low fluid intake increase stone risk?
104. Is stone disease more prevalent in hot climates?
105. In which occupational group is stone disease more common?

*0 = Incorrect or misleading ( The answer is factually wrong*,* misleading*,* or does not align with the guideline.)*,* 1 = Partially correct ( The answer includes some correct elements but lacks completeness*,* clarity*,* or accuracy.)*,* 2 = Fully correct ( The answer is fully accurate*,* complete*,* and clearly reflects the guideline recommendation.)*
